# Supporting the massive scale-up of antiretroviral therapy: the evolution of PEPFAR-supported treatment facilities in South Africa, 2005-2009

**DOI:** 10.1186/1471-2458-12-173

**Published:** 2012-03-09

**Authors:** Elysia Larson, Heidi O'Bra, JW Brown, Thobile Mbengashe, Jeffrey D Klausner

**Affiliations:** 1 American Schools of Public Health, U.S. Centers for Disease Control and Prevention, Pretoria, South Africa; 2 U.S. Centers for Disease Control and Prevention, PO Box 9536, Pretoria 0001, South Africa; 3 USAID, 100 Totius Street, Pretoria 0181, South Africa; 4 National Department of Health, Private Bag X828, Pretoria 0001, South Africa

**Keywords:** South Africa, HIV/AIDS, Health Systems, PEPFAR

## Abstract

**Background:**

South Africa has an estimated 1.5 million persons in need of antiretroviral therapy (ART). In 2004, the South African government began collaborating with the United States President's Emergency Plan for AIDS Relief (PEPFAR) to increase access to ART. We determined how PEPFAR treatment support changed from 2005-2009.

**Methods:**

In order to describe the change in number and type of PEPFAR-supported ART facilities, we analyzed routinely collected program-monitoring data from 2005-2009. The collected data included the number, type and province of facilities as well as the number of patients receiving ART at each facility.

**Results:**

The number of PEPFAR-supported facilities providing ART increased from 184 facilities in 2005 to 1,469 facilities in 2009. From 2005-2009 the number of PEPFAR-supported government facilities increased 10.1 fold from 54 to 546 while the number of PEPFAR-supported NGO facilities (including general practitioner and NGO facilities) increased 6.2 fold from 114 to 708. In 2009 the total number of persons treated at PEPFAR-supported NGO facilities was 43,577 versus 501,089 persons at PEPFAR-supported government facilities. Overall, the median number of patients receiving ART per site increased from 81 in 2005 to 136 in 2009.

**Conclusions:**

To mitigate the gap between those needing and those receiving ART, more facilities were supported. The proportion of government facilities supported and the median number of persons treated at these facilities increased. This shift could potentially be sustainable as government sites reach more individuals and receive government funding. These results demonstrate that PEPFAR was able to support a massive scale-up of ART services in a short period of time.

## Background

South Africa has the highest number of HIV infected individuals, and one of the highest prevalences of HIV infection in the world. In 2005 an estimated 4.7 million persons were HIV infected and only approximately 123,000 were receiving treatment [1]. In 2004, the South African government (SAG) began collaborating with the United States President's Emergency Plan for AIDS Relief (PEPFAR) to increase access to antiretroviral therapy, as well as other HIV care and prevention services. PEPFAR support was provided through the development of policies and guidelines and the provision of training, drugs, laboratory services, staff, equipment and technical assistance. By 2010 the U.S. government, through PEPFAR, had contributed over 2.4 billion U.S. dollars to address HIV/AIDS and tuberculosis in South Africa [2].

Effective scale-up of antiretroviral therapy delivery requires host country leadership and coordination between donors, implementing partners and host country governments. The initial emergency phase of PEPFAR implementation from 2004-2009 was through implementing partners, primarily non-governmental international, and local organizations, with the goal of rapidly increasing access to treatment to those most in need [3]. These non-governmental organizations work both in private and public sites, however, the SAG directed these early services toward public hospitals and large public clinics in order to quickly treat the largest and most accessible patient populations [4].

Facilities supported by PEPFAR can be broadly categorized as: public sector sites, non-governmental organization (NGO) sites, and general practitioner (GP) networks. In these facilities, PEPFAR implementing partners provide a range of support depending on the needs of the facility. All facilities that are counted in this review have PEPFAR-supported activities that are directly connected to service delivery at that site [5]. In public facilities, this support can include a combination of staff, equipment, training, information management and other activities. Support at non-governmental sites largely focused on service delivery through existing infrastructure managed by faith-based organizations. Support for GP networks included providing ART access to persons who were in the queue at public facilities or were uninsured or underinsured teachers or union workers. This report describes how PEPFAR support evolved at the treatment delivery (facility) level from October 2005 through September 2009.

## Methods

Since 2005 all PEPFAR implementing partners funded to support antiretroviral therapy have submitted quarterly monitoring data through a web-based system (MySQL, MySQL AB, Uppsala, Sweden) [6]. From October 2005 through September 2009 the reported indicators included persons currently on antiretroviral therapy, facilities providing antiretroviral therapy, the type of facility providing antiretroviral therapy (government, NGO, and private), and the province in which the facility operates. NGO partners may provide support to government facilities. Where data on management type and facility were missing, we contacted the projects' managers to complete the data. Twenty partners reported aggregate data for at least some of the facilities (for example one partner aggregated all the private general practitioner facilities) therefore not allowing us to determine location for that specific facility.

Some partners reported facilities as providing antiretroviral therapy, but then reported zero persons on antiretroviral therapy for that facility. We report the number of facilities in that category, but removed them from all analyses of facility size. For the reported aggregate data, the average facility size was determined by calculating the number of persons on treatment divided by the number of facilities. In instances where the quarterly report for a facility reported patients on treatment, but not the number of facilities, we recorded it as one facility (n = 42 facilities in 2005; n = 32 facilities in 2009).

We conducted a retrospective review of routinely collected PEPFAR monitoring data from September 2005 to October 2009. We describe the PEPFAR-supported sites by year, management type, location, and number of persons currently on treatment (reporting medians and ranges.) We describe the rate of change in number of facilities.

## Results

The number of PEPFAR-supported facilities providing antiretroviral therapy increased from 184 facilities in 2005 to 1,469 facilities in 2009, an average increase of 132.3 facilities each year. The median number of patients receiving antiretroviral therapy per site increased from 81 patients (range 3 to 1,584) in 2005 to 136 patients (range 1 to 8,656) in 2009. From 2005 to 2009 the number of non-government facilities (including private general practitioner and NGO facilities) increased 6.2 fold from 114 to 708 while the number of PEPFAR-supported government facilities increased 10.1 fold from 54 to 546 (management type was not recorded for 16 facilities in 2005 and 215 in 2009.) The number of persons treated at these facilities is shown in Table 1.

**Table 1:** The total number of persons treated at PEPFAR-supported facilities: October 2005, September 2009

	2005				2009			
	Total Facilities	Total Persons	Median patients per facility (Range)		Total Facilities	Total Persons	Median patients per facility (Range)	
**Non-governmental**	114	9,732	29	(3 - 797)	708	43,577	3	(1 - 4,112)
**NGO**	30	6,333	138	(27 - 797)	94	34,299	81	(1 - 631)
**Private**	84	3,399	13	(3 - 481)	614	9,278	3	(5 - 4,112)
**Governmental**	54	41,460	441	(25 - 4,332)	546	501,089	488	(4 - 8,656)

**Figure 1:**
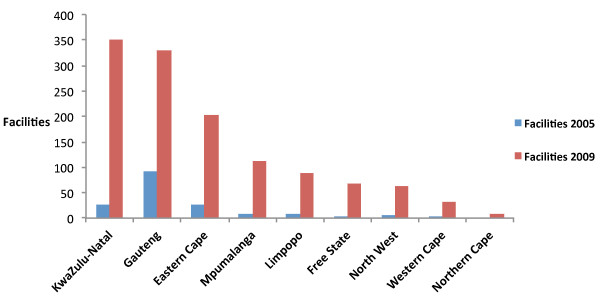
**Distribution of PEPFAR-supported facilities in South Africa, by province October 2005, September 2009**.

**Figure 2:**
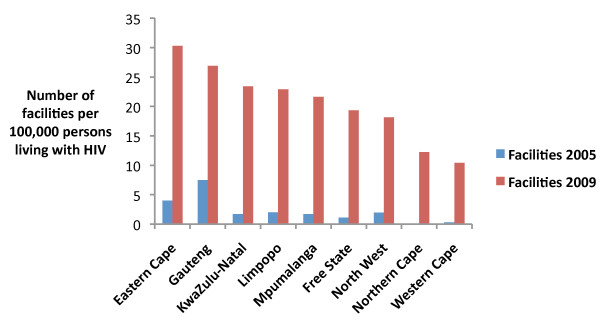
**Distribution of PEPFAR-supported facilities in South Africa standardized by number of persons living with HIV in 2008, by province October 2005, September 2009**.

There was a shift in the distribution of facilities by province from 2005 to 2009 (Figure 1.) When the distribution of facilities is standardized by the number of persons living with HIV, this shift is still apparent (Figure 2.) In 2005 there were 3.4 times as many PEPFAR-supported facilities in Gauteng as in any other province. In 2009 the province with the most PEPFAR-supported facilities was KwaZulu-Natal with 351 facilities, followed by Gauteng and Eastern Cape with 329 and 202 PEPFAR-supported facilities, respectively. Northern Cape had the fewest facilities with only 10.

In 2005 107 sites reported providing antiretroviral therapy services, but no persons currently on treatment versus only 28 such sites in 2009.

## Discussion

Through our analysis of routinely collected PEPFAR monitoring data we found that in order to support the provision of antiretroviral treatment to a increasing number of people in need in South Africa, new governmental and nongovernmental facilities were rapidly capacitated to provide treatment. Despite health system constraints and the SAG's initial reluctance to show full support for a national ART program, [7,8] the proportion of government facilities supported increased dramatically, as did the number of persons treated at governmental facilities.

The flexibility of PEPFAR to partner with both the private and public sector allowed for an initial engagement with the private sector, as it was equipped to assist in a quick roll-out of access to ART, followed by a transition to supporting a larger proportion of governmental facilities. Because governmental sites reach more individuals and are supported by the South African government scale-up of ART, supporting governmental sites may have a larger impact and be more sustainable. Flexibility in the scale-up of supported facilities allowed for this shift in the proportion of PEPFAR-supported sites with recorded management type from 32% public in 2005 to 44% public in 2009. While there were over 700 non-governmental facilities supported, about 65% were general practitioner sites with fewer than 10 patients. Additionally, the increase in number of persons treated at governmental sites indicates not only a PEPFAR ART scale-up, but also reflects the SAG's expanded commitment to universal access to treatment. The reduction in the median number of patients per facility in private facilities may indicate that as services become available in public facilities, people are less reliant on private facilities for treatment. Our findings are consistent with previous estimations of the number of persons on treatment by facility type, which showed that beginning in 2005 more people were on treatment through public sector sites than NGO or private and also that the increase in persons on treatment was more rapid in the public sector than non-governmental or private [9].

From 2005 to 2009 the geographic distribution of facilities continued to be focused in areas with high numbers of persons living with HIV, but also shifted to increase coverage across all provinces, suggesting a move toward deliberate scale up [2]. KwaZulu-Natal is the province with the largest number of persons living with HIV (approximately 1,489,972 persons in 2009), followed by Gauteng and Eastern Cape (approximately 1,132,901 and 674,420 persons respectively in 2009) [10,11].

The number of facilities may have been underestimated, as some implementing organizations did not report the number of facilities. However, most of these reports were hospitals, not clusters of facilities, indicating that they were indeed only one facility. Because almost one third of patients on treatment were reported from partners with aggregated facilities, the median size of the facilities may be skewed. In addition, the reduction over time in the number of facilities reporting that they provide treatment services, but not reporting persons currently on treatment, suggests an improvement in the quality of reporting.

The rapid scale-up of access to ART was a "game changer" in the fight against HIV & AIDS [12]. Treatment coverage has greatly increased, and was estimated to be about 85% in 2010 [1]. To continue providing assistance toward increasing coverage and maintaining treatment for many years to come, next steps will involve making the transition from an emergency oriented program to one that is focused on sustainability, on achieving cost efficiencies, and on full alignment with the SAG priorities and systems [13]. This will include transitioning from international to local implementing partners and increasing support for the SAG. PEPFAR technical assistance will increasingly emphasize health system strengthening, capacity development, quality of care, as well as innovative strategies such as nurse initiated and managed ART to provide sustainable, long-term access to care.

## Conclusions

With the delivery of health services occurring so frequently as a collaboration between donors, implementing organizations and governments it is essential that donors and implementing organizations be able to shift their support to meet the needs of host-country governments considering the epidemiology of the epidemic and current infrastructure. The South African government has greatly increased access to ART, mitigating the gap and promoting more equality Our analysis demonstrates that in order to support this effort, PEPFAR rapidly increased the number of facilities supported, particularly in areas with the highest prevalence of HIV.

## Competing interests

The authors declare that they have no competing interests.

## Authors' contributions

EL performed the analysis and drafted the manuscript. JWB, HOB, and TB provided programmatic insight and revised the manuscript. JDK conceptualized the analysis, edited the analysis, and revised the manuscript. All authors read and approved the final version.

## Funding

Support for this work was provided in part by the U.S. President's Emergency Plan for AIDS Relief (PEPFAR)

## Disclaimer

The findings and conclusions in this report are those of the authors and do not necessarily represent the official position of the institutions with which they are affiliated.

## Pre-publication history

The pre-publication history for this paper can be accessed here:

http://www.biomedcentral.com/1471-2458/12/173/prepub
